# Horse: a potential source of *Cryptococcus neoformans* and *Cryptococcus gattii* in Egypt

**DOI:** 10.1186/s12917-021-03127-x

**Published:** 2022-01-04

**Authors:** Rahma Mohammed, Sara M. Nader, Dalia A. Hamza, Maha A. Sabry

**Affiliations:** Department of Zoonoses, Faculty of Veterinary Medicine, Cairo University, PO Box 12211, Giza, Egypt

**Keywords:** *C. neoformans*, *C. gattii*, Horse, Serotyping, Egypt

## Abstract

**Background:**

Cryptococcosis is an opportunistic mycozoonosis of global significance in a wide variety of host species. In equines, cryptococcosis is uncommon, and sporadic cases have been reported with rhinitis, sinusitis, pneumonia, and meningitis. *Cryptococcus* spp. represents a potential risk for immunosuppressed and healthy persons. In Egypt, epidemiological data on cryptococcal infection in horses are limited. The current study was carried out to investigate the occurrence of *Cryptococcus* spp. in horses and its possible role in the epidemiology of such disease in Egypt.

A total of 223 samples was collected from different localities in Egypt included 183 nasal swabs from horses, 28 nasal swabs from humans, and 12 soil samples. Bacteriological examination and the identification of *Cryptococcus* spp. were performed. Molecular serotyping of *Cryptococcus* spp. was determined by multiplex PCR using CNa-70S/A-CNb-49S/A. The virulence genes (*LAC1*, *CAP59*, and *PLB1*) of the identified isolates were detected by PCR. Moreover, sequencing and phylogenetic analysis of the *C. gattii* gene from horses, humans, and soil isolates found nearby were performed.

**Result:**

The overall occurrence of *Cryptococcus* spp. in horses were 9.3, 25, and 10.7% in horses, the soil, and humans, respectively. Molecular serotyping of the *Cryptococcus* spp. isolates recovered from the nasal passages of horses proved that *C. gattii* (B), *C. neoformans*, and two hybrids between *C. neoformans* (A) and *C. gattii* (B) were identified. Meanwhile, in case of soil samples, the isolates were identified as *C. gattii* (B). The human isolates were serotyped as *C. gattii* in two isolates and *C. neoformans* in only one isolate. Molecular detection of some virulence genes (LAC1), (CAP59), and (PLB1) were identified in both *C. gattii* and *C. neoformans* isolates. The *C. gattii* gene amplicons of the isolates from horses, humans, and the soil were closely related.

**Conclusion:**

This study provides the first insights into the Egyptian horse ecology of *Cryptococcus* species and highlights the role of horses as asymptomatic carriers in disseminating the potentially pathogenic *Cryptococcus* spp. It also presents the possible risk of cryptococcosis infection in humans.

## Background

Cryptococcosis is a life-threatening systemic mycosis of global significance that affects a wide variety of host species. It is caused by the *Cryptococcus neoformans* species complex and the *Cryptococcus gattii* species complex and is usually associated with pulmonary and systemic infections in humans and animals [1, 2].

The *C. neoformans* species complex includes two separate species, *C. neoformans* (serotype A) represented by genotypes VNI, VNII, and VNB and *C. deneoformans* (serotype D) represented by genotype VNIV. The *C. gattii* species complex has five cryptic species with serotypes B and C and genotype from VGI to V. Moreover, interspecies hybrids between *C. neoformans* and *C. gattii* have been detected [3–5]. *C. neoformans* (A) is found worldwide, whereas *C. gattii* (B) has been restricted geographically to tropical and sub-tropical regions [1]. *C. gattii* has recently been identified in temperate climates, which suggests an ecological shift that is possibly associated with global temperature and moisture changes [6, 7].

Cryptococcosis has been described in a wide variety of host species, including cats, dogs, ferrets, llamas, porpoises, birds, and horses, affecting many, and often multiple, organ systems [8]. Furthermore, it is a classical result of interactions between the host-pathogen and environmental sources that comprise soil contaminated with avian (most often pigeon) guano (*C. neoformans*) or soil debris and decaying trees like eucalyptus trees (*C. gattii*) [9].

There is evidence to prove that humans and animals usually get the infection from environmental sources by inhaling fungal cells [10]. The human fungal pathogen *Cryptococcus* is able to rapidly and effectively adjust to variable conditions, favoring its survival in the environment and in the infected host [11]. The survival mechanisms associated with virulence in this opportunistic pathogen include the polysaccharide capsule, the melanin within the cell wall, urease and laccase enzymatic activities, the ability to grow at temperatures of up to 37 °C, and the production of degradable enzymes such as phospholipase B (PLB1) [12]. These virulence factors confer a selective advantage to *C. neoformans* that enables it to reside both in the environment and in mammalian hosts.

*C. neoformans* is the most common fungal pathogen that infects the human central nervous system [13]. *C. neoformans* causes life-threatening infections as meningoencephalitis primarily in immunocompromised hosts (immunodeficiency generally associated with AIDS) [14]. Unlike *C. neoformans*, *C. gattii* causes meningoencephalitis and pulmonary infections mainly in immunocompetent hosts.

In horses, the distribution of cryptococcosis is worldwide, whereas the majority of clinical infections are reported from Western Australia and Vancouver Island, British Columbia, Canada [8, 15]. Moreover, a fatal case of a disseminated *C. gattii* infection was identified in an Arabian horse imported from South Africa into the United Arab Emirates and identified as *C. gattii VGII*, which was closely related to another *C. gattii VGII* isolate from the Middle East [16].

In Egypt, most studies at the beginning of the year 2003 reported that the major subtype of the *C. neoformans* spp. complex isolated from either environmental (Plant or Bird origin) or clinical samples (buffaloes, cattle, sheep, and chicken) is *C. neoformans* (serotype A) [17, 18]. Besides, Elhariri et al. [19] revealed the identification of *C. neoformans* and *C. gattii* from pigeon dropping. Furthermore, plants from Qutur and Tanta area carried *C. gattii* [20]. Abdel-Salam [21] reported on the characterization of *Cryptococcus* serotypes A and A/D in clinical specimens (serum, CSF, and stool samples). But to date, little epidemiological data are available among cryptococcal infection in horses and humans in contact in Egypt.

Therefore, the current study was carried out to determine the prevalence of *Cryptococcus* spp. among horses and the role played by horses in the distribution of such pathogens in the surrounding environment and humans occupationally in contact with such animals.

## Methods

### Sample collection and preparation

A total of 223 samples were collected from different localities in Egypt (The Cairo, El-Fayoum, Qalyubia, and Giza Governorates) over a period of 1 year from December 2018 to December 2019. These samples included 183 nasal swabs from horses and 28 nasal swabs from humans. In addition, 12 soil samples were collected from the houses of horses. Data collected from each human and animal included age, gender, and underlying health problems.

The nasal swabs were taken using sterile bacteriological swabs under complete antisepsis conditions and inserted into both nasal vestibules and rotated vigorously. The collected samples were immediately transported in ice boxes to the laboratory and then inoculated into sterile Sabouraud dextrose broth (Oxoid) supplemented with chloramphenicol (0.1 g/L) (HiMedia).

Soil samples were collected in sterile plastic bags. The samples were prepared by mixing about 3–5 g of each sample into sterile test tubes containing 10 ml of Sabouraud dextrose broth supplemented with chloramphenicol. The tubes were shaken vigorously by vortex and allowed to stand for about 15 min [18].

### Isolation and phenotypic identification of *Cryptococcus* spp.

The inoculated swab samples of humans and horses in addition to the prepared soil samples were incubated at 37 °C for 24 h according to Horta et al. [22]. The supernatant of the incubated sample was streaked onto plates of SDA with chloramphenicol and then incubated at 37 °C for 48–72 h. The typical *Cryptococcus* colonies with a mucoid appearance were selected and identified by the microscopic morphology of yeast cells.

*Cryptococcus* isolates were confirmed based on melanin synthesis (brown color effect) by streaking on the tobacco agar plates [23]. The identification of *Cryptococcus* isolates was done using the Rapid yeast plus system (Remel, USA) [24].

### Extraction of the genomic DNA

Genomic DNA from the pure *Cryptococcus* isolates was extracted using the boiling method according to Mohammadi et al. [25]. The extracted DNA was stored at − 20 °C until further use.

### Molecular serotyping of *Cryptococcus* spp. isolates

Multiplex PCR was carried out to detect *C. neoformans* serotype A and *C. gattii* serotype B as previously described by Aoki et al. [26] using specific oligonucleotide primers set. PCR was carried out on a total volume of 25 μl, containing 3 μl of template DNA from each isolate, 12.5 μl of EmeraldAmp MAX PCR Master Mix (Takara, Japan), 0.5 μl of each primer (10 pmole/μl; Metabion, Germany), and PCR-grade water, all of which added up to 25 μl. The PCR products were visualized on 1.5% agarose gel.

### Molecular detection of the virulence genes (LAC1, CAP59, and PLB1)

Conventional PCR to amplify the *LAC1*, *CAP59*, and *PLB1* genes was performed according to the method of Meyer et al. [27]. Briefly, the amplifications were carried out in a total volume of 25 μl, containing 3 μl of template DNA from each isolate, 12.5 μl of EmeraldAmp MAX PCR Master Mix (Takara, Japan), 0.5 μl of each primer (1 μM) (Metabion, Germany), and completed up to 25 μl by PCR-grade water. The PCR amplicons of the three genes were electrophoresed on 1.5% agarose gel.

### Sequencing and sequence analysis

The amplification products of three *C. gattii* isolates from horses, humans, and the soil were selected and purified using the QIAquick gel extraction kit (QIAGEN, Germany) according to the manufacturer’s instructions and sequenced at the Animal Health Research Institute (Giza, Egypt) using the forward and reverse primers of *C. gattii* (CNb-49 s and CNb-94A).

The nucleotide sequences have been deposited in the National Center for Biotechnology Information (NCBI) GenBank database under the accession numbers MT520167-MT520169.

The nucleotide sequences of *C. gattii* isolates were compared with the sequences available in the public domain using the NCBI BLAST server. Publicly available gene sequences were retrieved from the NCBI GenBank and aligned using CLUSTALW in BioEdit version 7.0.1.4. Phylogenetic analysis was performed with MEGA version X using the neighbor-joining approach. The bootstrap consensus tree was estimated from 1000 replicates.

## Results

The overall occurrence of *Cryptococcus* spp. collected from 183 nasal swabs of horses at different localities in Egypt was 17/183 (9.3%). Molecular serotyping of the *Cryptococcus* spp. isolates recovered from the nasal passage of horses proved that *C. gattii* B was identified in 12 isolates and *C. neoformans* A in 3 isolates. Two hybrids between *C. neoformans* A and *C. gattii* B were also identified. In the case of soil samples, *C. gattii* B was detected in 3 out of 12 samples (25%) collected from horse houses. The human isolates were serotyped as *C. gattii* in two isolates (7.1%) and *C. neoformans* in only one isolate (3.6%).

The detection rates of *Cryptococcus* spp. in healthy horses showed that *C. gattii* was identified in 5 out of 44 samples (11.4%). In the case of diseased horses, the detection rate was 12/139 (8.6%), and the identified *Cryptococcus* spp. in each case mentioned in (Table 1). The occurrence of the *Cryptococcus* spp. was nearly similar in both males and females as shown in Table 1.

**Table 1:** Occurrence of *Cryptococcus* spp. in horses according to health condition and gender

Predisposing factors	No of isolates		***Cryptococcus*** spp identified			% of positive isolates
**Health condition**						
Healthy	5		*C. gattii* (5)			11.4 (5/44)
Diseased	12	Respiratory tract signs (6)	*C. gattii* (5)	*C. neoformans* (1)		8.6 (12/139)
		Nervous Signs (4)	*C. gattii* (2)	*C. gattii* & *C. neoformans* hybrids (2)		
		Wounds treated with long course of antibiotics (2)	*C. neoformans* (2)			
**Gender**						
Male	11		*C. gattii* (8)	*C. neoformans* (1)	*C. gattii* & *C. neoformans* hybrids (2)	9.5 (11/116)
Female	6		*C. gattii* (4)	*C. neoformans* (2)		9 (6/67)
**Total**	**17**					**9.3 (17/183)**

Regarding the human samples, *Cryptococcus* spp. were identified from 3 out of 28 samples (10.7%), and *C. neoformans* serotype (A) was isolated from the nasal passage of an immune-compromised male suffering from chest allergy. In addition, *C. gattii* was isolated from two asymptomatic individuals. The positive samples belonged to humans working in stud farms where the horses’ positive samples were collected. Our results indicated that *Cryptococcus* spp. was more prevalent in old male individuals. Moreover, we found that *Cryptococcus* spp. more frequently colonized the nasal passages of smokers than those of non-smokers as shown in Table 2.

**Table 2:** Occurrence of *cryptococcus* spp. in human according to some risk factors

Risk Factors	Number of samples	Number of positive samples	% of positive samples
**Age (year)**			
30–40	19	2	10.5
41–50	5	0	0
> 50	4	1	25
**Gender**			
Male	27	3	11.1
Female	1	0	0
**Health Condition**			
Healthy	20	2	10
Diseased	8	1	12.5
**Smoking**			
Smoker	13	2	15.4
Non-smokers	15	1	6.6
**Total**	**28**	**3**	**10.7**

Diseased persons suffer from (diabetes and chest allergy, par nasal sinusitis and cough)

**Fig. 1:**
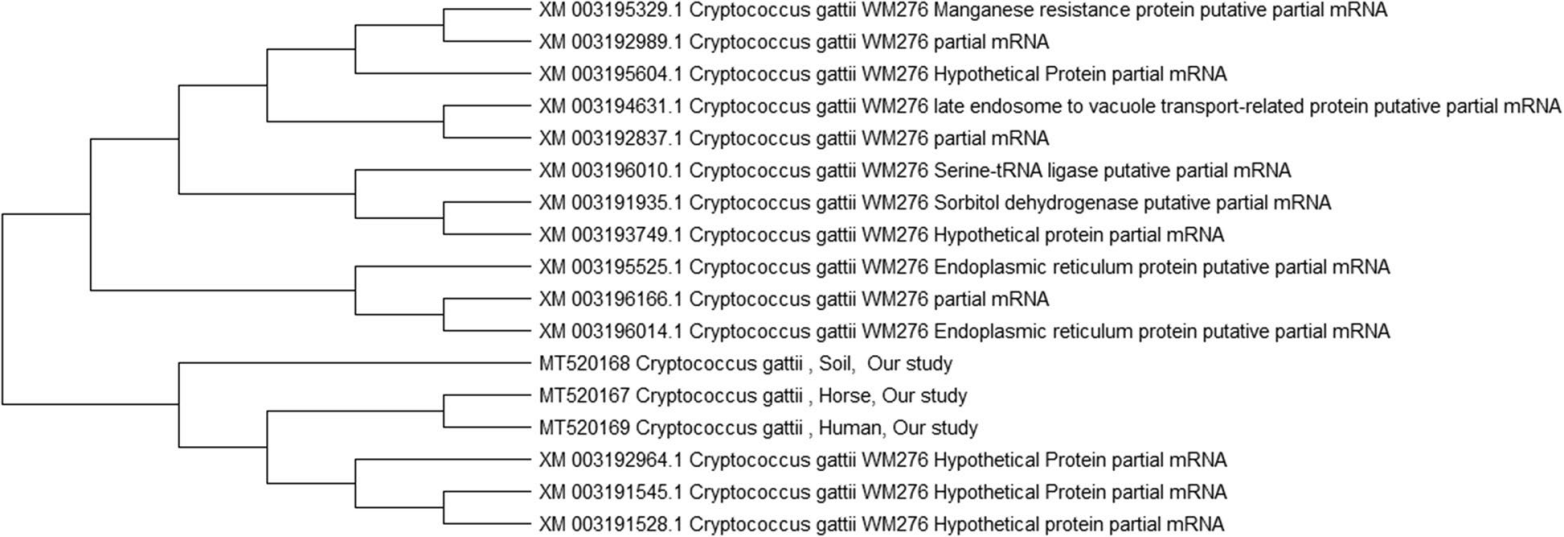
Phylogenetic analysis using the neighbor-joining method based on the partial sequence of *C. gattii* (CNb 49 S-A). Phylogenetic analysis was performed with MEGA version X using the neighbor-joining approach. The bootstrap consensus tree was estimated from 1000 replicates

The molecular detection of some virulence genes *LAC1*, *CAP59*, and *PLB1* was carried out in both *C. gattii* and *C. neoformans* isolates (Table 3). Comparing the partial sequences of *C. gattii B* revealed homology between the three selected isolates from horses, the soil, and humans in the same vicinity (Fig. 1).

**Table 3:** Serotyping and virulence pattern of *cryptococcus* spp. Recovered fom horse, human and soil samples

Sample	Isolates number	Serotypes	Virulence pattern
**Horses**	**17**	**(12) *C. gattii* Serotype (B)**	Positive for (LAC1, CAP59, PLB1)
		** (3) *C. neoformans* Serotype (A)**	
		**(2) Hybrid between* C. neoformans* and* C. gattii* (AB)**	
**Human**	**3**	**(2) *C. gattii* Serotype (B)**	
		**(1) *C. neoformans* Serotype (A)**	
**Soil**	** 3**	**(3) *C. gattii* Serotype (B)**

## Discussion

The published papers clarified the problem of mycoses in horses, including pathogenic fungi, from different parts of the world [28]. Among the mycotic diseases of horses, Cryptococcosis is of particular concern [29]. Egypt is one of the countries widely known for breeding Arabian horses for the local and foreign markets. In addition, the horse populations in Egypt are widely used in tourism [30]. However, up to date, there have not been enough epidemiological data in Egypt for cryptococcal infection in horses.

In the current study, the overall recorded prevalence of *Cryptococcus* spp. colonization detected in the nasal passages of the examined horses was 9.3%, with the value being higher (11.4%) in healthy examined horses, which probably indicates that apparently healthy horses could be asymptomatic carriers of *Cryptococcus* spp. These findings are supported by those of Malik et al. [31], Connolly et al. [32], Duncan et al. [33], and Mohamed et al. [34], who isolated *Cryptococcus* spp. from the nasal passages of dogs, cats, koalas, and donkeys without any evidence of disease, suggesting the asymptomatic colonization of the nasal mucosa following environmental exposure.

The species identification of *Cryptococcus* appeared to be higher with *C. gattii* (12 isolates) in examined horses than with *C. neoformans* (3 isolates). This result was similar to that of Mcgill et al. [15], who detected *Cryptococcus* spp. from 20 out of 155 affected horses in Western Australia with a high frequency of *C. gattii*, and Duncan et al. [8], who identified *C. gattii* in the nasal passage of horses in Canada.

Hybrids between *C. neoformans* (A) and *C. gattii* (B) were also identified in two isolates, which is in line with the findings of Bovers et al. [35] and Aminnejad et al. [4], who reported that *C. gattii* is capable of forming hybrids with *C. neoformans*. Interspecific cryptococcal hybrids have been reported earlier in Germany [36], Denmark [37], and the United States [38]. Inter-varietal and interspecies hybrids of *Cryptococcus* are more virulent and cause mammalian diseases [39]. This hybrid might become a “superpathogen,” which has a worldwide distribution like *C. neoformans* and is able to infect immunocompetent humans like *C. gattii*, combining the malicious characteristics of both species [35].

The pathogenic species *C. neoformans* and *C. gattii* infect immune-suppressed and immune-competent individuals, respectively [1, 40]. However, the data on the asymptomatic isolation of *Cryptococcus* spp. from human nasal passages are very rare. In this study, we isolated *Cryptococcus* spp. from people without cryptococcosis, Morera et al. [41] supported similar findings of the asymptomatic nasal carriage of *C. gattii* in humans sharing the same house with domestic ferret having cryptococcosis.

The occurrence of *Cryptococcus* spp. in the nasal passage of humans who have occupational contact with infected horses was 10.7% (3 out 28); two of them were reported in aged individuals who were smokers, which is in line with the findings of Forthal et al. [42] and Pourbaix et al. [43], who reported that smoking was a risk factor for invasive fungal diseases such as cryptococcosis.

In this study, the *C. neoformans* serotype A was identified from the nasal passage of an immune-compromised male suffering from chest allergy. In addition, the *C. gattii* isolate was isolated from two asymptomatic individuals in contact with horses. In this context, a clinical isolate was obtained from an immigrant worker from Kuwait that is closely related to a case of *C. gattii* in an Arabian horse in the United Arab Emirates [16].

The epidemiological significance of this research lies in its proving that both horses and humans are at risk of environmental contamination in stud farms. In these farms, eucalyptus trees are used for horse shelter and shadowing [44], and these trees are one of the sources of *C. gattii*. Besides, the pigeon is a natural reservoir of *C. neoformans*, and it usually eats the remains of equine food (e.g., seeds and grains) from the feed troughs and defecates in the feed and water trough.

To support our epidemiological view, the soil around horses included in this research was examined for *Cryptococcus* spp. and the result revealed 25% from which all isolates were *C. gattii*. This may be attributed to the fact that that soil contaminated with plant debris and decaying wood is a major environmental source of *C. gattii* [7].

The isolation of *C. gattii* from the nasal passage of horses, humans, and soil samples in close vicinity to the examined horses point to the presence of a common issue between these isolates, which was predicted to be the excessive presence of eucalyptus trees in the collection sites. This result is in line with that of Mcgill et al. [15], who reported that horses were more likely to be infected with *C. gattii* than with *C. neoformans*, and this was attributed to horse population’s close association with eucalyptus trees, a regional environmental reservoir of *C. gattii*.

Human cryptococcosis develops following environmental exposure and inhalation of the infectious spores of *Cryptococcus* spp. This is enhanced by some virulent determinants, which are enzyme secretion, the addition of melanin to the fungal cell wall, and the abundant secretion of polysaccharides that may surround the cell to form a capsular structure [45].

In this study, we investigated the presence of some virulence genes such as laccase (*LAC1*), capsular-associated gene (*CAP59*), and phospholipase (*PLB1*) in the identified *Cryptococcus* spp. isolates. The International Society of Human and Animal Mycology (ISHAM) group selected the Multilocus Sequence Type (MLST) analysis as the method of choice for global molecular epidemiological typing of *Cryptococcus* species using seven housekeeping loci (CAP59, GPD1, LAC1, PLB1, SOD1, URA5, and IGS1) [27]. The study identified the laccase (*LAC1*) gene, which is the main protein involved in the production of the melanin pigment, in all *C. neoformans* and *C. gattii* isolates [46, 47]. The study also demonstrates the presence of the *CAP59* gene in the identified isolates. This capsular gene has a vital role in *Cryptococcus* virulence. Robertson et al. [48] demonstrated that strains producing large capsules were more likely be associated with higher intracranial pressures and lower fungal clearance rates upon treatment with amphotericin B and fluconazole in HIV-infected patients. Furthermore, we identified the phospholipase (PLB1) gene (which is an important gene in maintaining the integrity of the chitinous fungal cell wall as well as providing nutrients that can be utilized as a carbon source for *C. neoformans* during the course of the infection) in all isolates [49].

The studied virulence genes have been generally found to be recovered from the horses, horses’ environments, and humans in contact with the horses, which is in line with the findings of Firacative et al. [50], who supports the theory of the acquisition of the infection from environmental sources. Likewise, Esher et al. [51] hypothesized that the virulence traits were acquired for survival in the environment and then re-purposed in the setting of mammalian infection.

In the present study, a phylogenetic relationship was required to predict the epidemiological infection cycle of *Cryptococcus*. *C. gattii* sequenced from different sources (humans, horses, and the soil) confirmed that it can be isolated from those three sources with the same sequence type and indicated that *C. gattii* was shared between humans, animals, and the environment. The phylogenetic tree showed that the three sequences were found on the same clade, which explains the probability of the transmission of the infection between these sources. The presence of eucalyptus trees in equine farms could be one of the environmental sources of infection of horses with *C. gattii*, which would explain how the people in the surroundings get infected. The same theory was put forward by Farrer et al. [5], who discovered *C. gattii* VGV in samples from Hyrax-associated environments, which is suggestive of an association with these mammals that can spill over into humans.

## Conclusion

The present study confirmed the presence of *C. neoformans* and *C. gattii* from clinical samples (nasal swab from humans and horses) and *C. gattii* from environmental samples (soil) with clarifications of the possible epidemiological relationship between environmental and clinical isolates. Further studies need to be carried out on virulence by measuring capsule size and enzyme production. The research results provide insights into the potential exposure risk of humans in contact with cryptococcal infection in the investigated locations.

## Data Availability

All the data generated or analyzed in this study are included in this published article.
